# Light, Sound, and Melatonin: Investigating Multisensory Pathways for Visual Restoration

**DOI:** 10.3390/medicina61061009

**Published:** 2025-05-28

**Authors:** Dario Rusciano

**Affiliations:** Fidia Ophthalmic Research, 95123 Catania, Italy; drusciano55@gmail.com

**Keywords:** multisensory integration, ipRGC, melanopsin, superior colliculus, crossmodal plasticity, melatonin, visual rehabilitation, blindsight

## Abstract

Multisensory integration is fundamental for coherent perception and interaction with the environment. While cortical mechanisms of multisensory convergence are well studied, emerging evidence implicates specialized retinal ganglion cells—particularly melanopsin-expressing intrinsically photosensitive retinal ganglion cells (ipRGCs)—in crossmodal processing. This review explores how hierarchical brain networks (e.g., superior colliculus, parietal cortex) and ipRGCs jointly shape perception and behavior, focusing on their convergence in multisensory plasticity. We highlight ipRGCs as gatekeepers of environmental light cues. Their anatomical projections to multisensory areas like the superior colliculus are well established, although direct evidence for their role in human audiovisual integration remains limited. Through melanopsin signaling and subcortical projections, they may modulate downstream multisensory processing, potentially enhancing the salience of crossmodal inputs. A key theme is the spatiotemporal synergy between melanopsin and melatonin: melanopsin encodes light, while melatonin fine-tunes ipRGC activity and synaptic plasticity, potentially creating time-sensitive rehabilitation windows. However, direct evidence linking ipRGCs to audiovisual rehabilitation remains limited, with their role primarily inferred from anatomical and functional studies. Future implementations should prioritize quantitative optical metrics (e.g., melanopic irradiance, spectral composition) to standardize light-based interventions and enhance reproducibility. Nonetheless, we propose a translational framework combining multisensory stimuli (e.g., audiovisual cues) with circadian-timed melatonin to enhance recovery in visual disorders like hemianopia and spatial neglect. By bridging retinal biology with systems neuroscience, this review redefines the retina’s role in multisensory processing and offers novel, mechanistically grounded strategies for neurorehabilitation.

## 1. Introduction

The integration of multiple sensory signals arises through complementary systems: neural convergence in the brain and specialized circuits in peripheral structures. While these systems operate via distinct mechanisms and pathways, they are dynamically interconnected. For example, peripheral sensory organs like the retina (e.g., via intrinsically photosensitive retinal ganglion cells, ipRGCs) not only relay signals but also modulate central multisensory processing, creating feedback loops that shape perception. This interplay between early sensory modulation and higher-order integration underscores the importance of studying multisensory interactions as a continuum—from peripheral preprocessing to cortical synthesis. This interdependent framework provides a foundation for examining how perturbations at any level—whether peripheral or central—can spread through cross-sensory pathways and vision–brain connections, changing how we perceive the world.

### 1.1. Multisensory Integration in the Brain

Our brains seamlessly blend sight, sound, and touch into unified experiences, even when these signals are not perfectly aligned [1]. This multisensory integration relies on a sophisticated neural dialogue between lower and higher brain regions [2]. Subcortical structures like the superior and inferior colliculi (SC, IC) receive direct sensory inputs and serve as initial integration hubs [2], relaying information to cortical regions where more complex blending occurs through synchronized electrical activity [3].

What makes this system remarkable is its dynamic flexibility. Higher brain areas do not just process incoming signals—they continuously generate predictions about what sensory inputs to expect [2,3]. When mismatches occur (like when a sound arrives slightly before its corresponding visual event), the brain automatically computes which signal is more reliable using probabilistic weighting strategies [4]. This Bayesian-like computation enables the brain to resolve conflicts, automatically adjusting the weighting of each sensory modality [1,4,5]. The entire process is synchronized by rhythmic electrical activity that coordinates communication across brain areas. This creates coherent perception despite imperfect alignment, explaining everyday experiences like understanding speech in noisy environments or coordinating precise movements [5]. The entire system operates through continuous, bidirectional communication that maintains perceptual stability while accommodating sensory variability [2,3].

### 1.2. Specialized Sensory Circuits in Peripheral Structures

Our brain’s ability to combine information from different senses follows an organized pathway from initial detection to conscious perception and action. This process begins at the sensory organs, where specialized receptors transform physical stimuli into neural signals—photoreceptors in the eyes convert light, hair cells in the ears transduce sound, and various receptors in the skin detect touch [6]. These signals travel via the peripheral nervous system to the brainstem, where early integration occurs [7].

### 1.3. Where Integration Occurs

The SC combines visual, auditory, and somatosensory inputs to enhance stimulus detection and localization (see Section 2 for detailed SC mechanisms). However, the extent to which cortical feedback (experience-dependent) vs. subcortical inputs dominate integration is actively debated [8]. The IC pre-processes auditory inputs for spatial mapping, while the thalamus relays modality-specific signals to cortical layers, regulating excitability [9].

In the cerebral cortex, multisensory integration occurs hierarchically yet across distributed networks. Parietal regions merge vision, touch, and proprioception for spatial perception, while primary sensory areas contribute to early crossmodal interactions (e.g., auditory/tactile modulation of visual processing within ~100–200 ms [10]). This multistage integration creates unified environmental percepts that guide actions like catching balls or avoiding obstacles [11].

When sensory conflicts arise, the brain engages a hierarchical resolution cascade: early visuospatial attention (indexed by sharpened alpha oscillations, 8–12 Hz) first enhances selectivity for task-relevant inputs, as demonstrated during incongruent multisensory processing [12]. Later, frontal executive processes (theta oscillations, 3–7 Hz) refine decisions based on stimulus salience and context [13], aligning with their role in conflict resolution.

Multisensory integration depends on feedback loops between cortical and subcortical areas. The SC requires corticotectal inputs to maintain integration [14], while thalamocortical projections reprogram sensory cortices based on input statistics [15]. These interactions operate across timescales—from fast online modulation (alpha/gamma oscillations) to slow developmental plasticity [16].

Multisensory training improves outcomes in visual deficits (e.g., hemianopia [17]), with the SC leveraging crossmodal plasticity (e.g., auditory facilitation of blind-field processing [18]). Patients with blindsight (residual vision after V1 lesions [18]) or neglect unconsciously use crossmodal cues to compensate for deficits, demonstrating adaptive conflict-resolution strategies.

Table 1 summarizes these pathways, highlighting the SC’s role as an audiovisual (AV) integration hub for sensory compensation [19].

The process of multisensory integration is not always perfect, and conflicts between sensory inputs often arise. To resolve these ambiguities, the brain employs adaptive strategies—weighing prior experience, attentional focus, and stimulus salience to determine the most reliable input [20]. These same mechanisms take on profound clinical significance in sensory rehabilitation. For instance, patients with hemianopia (including those exhibiting blindsight) unconsciously leverage crossmodal cues (like sounds or tactile signals) to compensate for visual field loss, demonstrating how conflict-resolution strategies can be harnessed to improve functional vision. This dynamic process not only allows healthy individuals to perceive the world accurately amid sensory noise but also enables neurological populations to adapt to sensory deficits, transforming fragmented inputs into coherent percepts that guide effective interaction with their environment.

NB: here, ‘blindsight’ refers specifically to residual visual capacities in cortically blind fields (e.g., after V1 lesions [17,18]), while ‘non-conscious detection’ encompasses broader implicit processing (e.g., in neglect or healthy subjects [3]).

## 2. The Superior Colliculus

### 2.1. Laminar Organization: From Sensory Input to Motor Output

The SC is a conserved midbrain structure that integrates visual, auditory, and somatosensory inputs to guide orienting behaviors, as demonstrated by decades of electrophysiological and lesion studies [2,21,22]. It orchestrates sensory–motor integration through laminar-specific processing. Its superficial layers are dominated by unisensory visual neurons that receive topographically organized retinal inputs, forming precise retinotopic maps to encode spatial features (direction, velocity, location) of visual stimuli. These maps directly guide reflexive gaze shifts via projections to brainstem saccade generators (e.g., paramedian pontine reticular formation) [21]. Beneath them, the intermediate and deep layers exhibit a functional transition: modality-specific auditory neurons (processing sound localization cues from the IC) and somatosensory neurons (responding to tactile/proprioceptive signals) intermingle with anatomically multisensory neurons.

### 2.2. Multisensory Integration in the SC: Principles and Mechanisms

These deeper-layer multisensory cells integrate spatially aligned crossmodal inputs (e.g., visual–auditory or visual–somatosensory cues), amplifying responses through nonlinear summation (“inverse effectiveness”). Weak unimodal inputs combine to produce supralinear output enhancements (300–500% magnitude increases), sharpening detection thresholds and reducing reaction times [2]. Anatomically, SC multisensory neurons are specialized for integrating spatially aligned AV cues [22], while crossmodal-responsive cortical areas (e.g., parietal) exhibit task-dependent modulation [10]. Critically, these deep layers also project to premotor and brainstem nuclei (e.g., reticulospinal tracts), converting integrated sensory signals into coordinated orienting movements. The system’s efficacy is dynamically tuned by corticotectal feedback (e.g., from anterior ectosylvian sulcus) and basal ganglia loops, which adjust integration weights based on attention and learned crossmodal statistics [21]. This layered architecture—from retinotopic visual specificity in superficial strata to sensorimotor transformation in deep strata—establishes the SC as an adaptive interface, where the interplay of unisensory precision and multisensory plasticity enables precise navigation of complex environments.

Table 2 illustrates different types of neurons in the superior colliculus, highlighting their roles in processing sensory information. In this review, ‘multisensory neurons’ refer to cells with anatomically confirmed convergent inputs (e.g., SC deep-layer neurons) unless specified otherwise. Functionally engaged neurons (e.g., cortical cells during AV tasks) are termed crossmodal responsive.

### 2.3. The SC as a Hub for Orienting and Defensive Behaviors

The SC anatomically multisensory neurons actively transform integrated inputs into behaviorally relevant signals. By synthesizing visual, auditory, and somatosensory cues, these neurons enable spatial orientation, coordinated eye–head movements (e.g., gaze shifts toward looming sounds), and reflexive defensive actions [2]. This integration is dynamically adjusted through corticotectal feedback and ascending modulatory inputs [21,27]. The SC’s plasticity supports functional recovery in hemianopia patients, where auditory cues enhance blind-field localization [18], and AV training improves oculomotor control—likely via SC-mediated spatial remapping [28]. While ipRGCs sustain SC excitability through melanopsin-driven inputs, their role appears distinct from classical multisensory convergence. Recent comprehensive mapping of SC circuits [27] shows that modulatory inputs (e.g., from cortical associative areas) tune SC zones without direct multisensory integration, and multisensory neurons (in SC.cm/SC.cl zones) receive convergent sensory inputs for sensorimotor transformation. This organizational dichotomy suggests ipRGCs may operate like corticotectal projections—enhancing SC responsiveness (e.g., to residual visual inputs) rather than participating in direct crossmodal summation. Such architecture underscores the SC’s adaptability for rehabilitation, where combined multisensory training and non-image-forming light stimulation could synergistically boost residual function.

### 2.4. Plasticity and Rehabilitation: Lessons from Hemianopia and Neglect

The clinical consequences of disrupted SC function underscore its necessity in both reflexive and volitional behavior. In hemispatial neglect—caused by lesions to the SC-parietal network—patients fail to perceive or respond to contralesional stimuli despite intact primary senses. For example, they may ignore a ringing phone on their left, even when the sound is loud [29]. This deficit reveals the SC’s dual roles: its reflexive pathways (via brainstem projections) orient gaze to sudden crossmodal cues, while its volitional circuits (through basal ganglia and frontal eye field (FEF) connections) integrate spatial attention with goal-directed action. This dichotomy mirrors the SC’s connections: its caudal deep layers project to brainstem saccade generators (e.g., inhibitory burst neurons in the parabigeminal nucleus) to trigger reflexive gaze shifts via omnipause neuron inhibition [30], while rostral intermediate layers communicate with the FEF via mediodorsal thalamus to align attention with intentions.

### 2.5. Clinical Insights: Reflexive vs. Volitional Circuits

Critically, multisensory interventions leverage this duality. Combined auditory–visual stimulation engages the SC’s reflexive orienting capacity, while prism adaptation—a therapy using optically shifted goggles to recalibrate spatial perception—recruits volitional circuits to remap coordinates [31]. These approaches synergistically exploit residual plasticity: reflexive SC-brainstem loops restore immediate responses to stimuli, while SC-cortical feedback gradually rewires spatial awareness. Together, these findings illustrate how the SC’s layered architecture—bridging automatic detection (deep layers) and adaptive behavior (intermediate-to-cortical pathways)—makes it indispensable for real-world navigation.

## 3. The Retina’s Hidden Role in Multisensory Perception—Melanopsin, ipRGCS, and Crossmodal Rehabilitation

**Figure 1:**
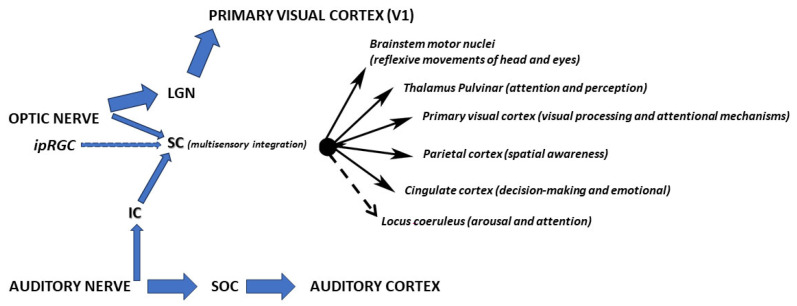
Pathways of audiovisual multisensory integration in the brain. Solid lines: empirically validated pathways. Dashed lines: hypothesized or inferred functional connections. LGN: lateral geniculate nucleus; SC: superior colliculus; IC: inferior colliculus; SOC: superior olivary complex.

The retina is often regarded as a mere gateway for visual perception, a neural canvas where photons are transformed into electrical signals destined for the visual cortex. Yet, emerging research reveals a far more complex and integrative role—one where retinal neurons, particularly ipRGCs, participate in multisensory processing, circadian regulation, and even cognitive functions. These cells, which express the photopigment melanopsin (encoded in mammals by the gene *OPN4*), function not merely as passive light detectors but as active participants in sensory integration, establishing direct connections with subcortical structures, including the superior colliculus (SC), suprachiasmatic nucleus (SCN), and lateral geniculate nucleus (LGN) [32,33]. RGCs serve as the retina’s primary output neurons, relaying both visual and non-visual information to diverse brain targets [34]. Among these RGCs, ipRGCs exhibit unique multimodal potential. While it is well established that ipRGCs, via melanopsin signaling, regulate circadian rhythms and pupillary reflexes [35,36], emerging evidence suggests broader integration capabilities. Strikingly, recent work demonstrates that ipRGCs can bypass canonical visual pathways entirely, driving behaviors like contagious itch via an SCN-thalamic circuit—independent of the SC or visual cortex [37]. Such findings underscore the retina’s role as a selective filter for biologically relevant stimuli, integrating light signals with broader physiological and behavioral demands. Moreover, ipRGCs receive modulatory inputs from neurotransmitters (e.g., norepinephrine) and hormones (e.g., melatonin), enabling them to encode internal states alongside environmental light [38,39]. Critically, melatonin’s influence on ipRGCs may be amplified by the presence of MT_1_ and MT_2_ receptors in the SC, targeted by ipRGC projections [40]. These receptors are localized to neuronal somata and dendrites in the SC, suggesting that melatonin could directly modulate SC excitability and, by extension, AV integration. This anatomical overlap positions ipRGCs as dual regulators of sensory and arousal systems: they relay ambient light signals via melanopsin while simultaneously integrating hormonal state information (e.g., circadian melatonin fluctuations) to fine-tune SC-dependent multisensory processing [2] (Figure 1). Despite these advances, important questions remain. While ipRGC projections to regions such as the parabrachial nucleus (for threat processing) and ventrolateral preoptic nucleus (for sleep regulation) have been identified [41], their potential role in crossmodal plasticity—particularly in rehabilitation paradigms for conditions like spatial neglect [17]—requires further investigation. This gap in knowledge presents a critical opportunity to bridge fundamental discoveries in ipRGC biology with clinical applications in sensory restoration.

Having established ipRGCs’ anatomical connections and modulatory potential, it is now interesting to investigate their functional capacity to link light perception with multisensory behavior. This exploration begins with their defining characteristic—melanopsin—the photopigment that enables their unique role as the retina’s multisensory integrators.

## 4. ipRGCs: Bridging Light Detection and Sensory Integration

### 4.1. Melanopsin and the Discovery of a Non-Classical Photoreceptor

For decades, vision was thought to rely solely on rods and cones. However, the discovery of melanopsin—a light-sensitive opsin expressed in a subset of retinal ganglion cells (ipRGCs)—upended this paradigm [42]. Unlike rods and cones, which hyperpolarize in response to light, ipRGCs depolarize, sending sustained signals to brain regions regulating circadian rhythms, pupillary reflexes, and even mood [35,41]. Critically, ipRGCs extend beyond non-visual functions. Their projections to the SC suggest they may influence spatial attention, gaze control, and crossmodal perception [43].

### 4.2. ipRGCs as Multisensory Modulators

While ipRGCs are primarily known for their role in circadian entrainment, their extensive connectivity suggests they may influence multisensory processing through indirect mechanisms. ipRGCs are capable of direct light detection, as melanopsin within these cells demonstrates peak sensitivity to blue light (~480 nm), enabling them to encode ambient illumination independently of traditional image-forming pathways [35]. Additionally, they receive inputs from rods and cones, allowing them to integrate both photopic (daylight) and scotopic (low-light) signals, thereby enhancing their responsiveness to a range of lighting conditions [44].

Emerging evidence indicates that ipRGC activity is modulated by various non-photic signals, which fine-tune their light-dependent responses rather than enabling direct multisensory integration. For instance, dopaminergic signaling influences the retinal circadian clock through melanopsin-dependent pathways [39], while serotonin accumulation in amacrine cells coupled to ipRGCs suggests neurochemical modulation of their activity [38]. Broader evidence indicates that serotonin contributes to light-dependent mood regulation [45], further highlighting how neurochemical systems may shape ipRGC signaling. Intriguingly, while ipRGCs are specialized for light detection, auditory cues can influence circadian rhythms through parallel pathways such as the intergeniculate leaflet (IGL) and serotonergic raphe nuclei projections, which converge with ipRGC outputs in the suprachiasmatic nucleus (SCN) [46,47].

Together, these findings position ipRGCs as dynamic regulators that adapt to both photic and non-photic cues, though they do not directly integrate multisensory inputs. Their projections to multisensory nuclei like the SC enable indirect crossmodal influences—for instance, SC neurons combine ipRGC-derived light signals with auditory inputs to guide orienting behaviors [27,43]. Thus, ipRGCs may act as contextual gatekeepers, modulating how and when light cues influence downstream multisensory processing—a role further tuned by circadian mechanisms (see below).

### 4.3. The ipRGC-Superior Colliculus Pathway: A Crossmodal Link

The SC orchestrates orienting behaviors by integrating multisensory cues (Section 1.3 and Section 2). ipRGCs fine-tune SC excitability through melanopsin signaling, particularly under low-light conditions [43,48], enabling rapid responses in degraded visibility [19,43]. While animal studies confirm this pathway’s role in gaze control [48], direct evidence for melanopsin-mediated SC modulation in human AV integration is lacking—a gap needing human electrophysiology or ipRGC-specific paradigms, such as recordings of ipRGC modulation of SC multisensory neurons. Nonetheless, by potentially optimizing the SC’s sensitivity to crossmodal stimuli, ipRGCs could enhance temporal alignment, a mechanism explored in rehabilitation for hemianopia or neglect (Section 5).

Critically, Stein & Rowland [19] demonstrate that repeated exposure to spatiotemporally congruent AV stimuli restores SC-mediated vision in hemianopic animals, adhering to the principles governing SC multisensory plasticity (e.g., input synchrony and spatial coincidence). Although direct evidence of melanopsin-mediated depolarization in SC neurons is lacking, ipRGCs project to the SC [48], and melanopsin activation has been shown to increase excitability in other subcortical targets (e.g., SCN, PVT) via sustained depolarization [49].

Neurocomputational models of SC plasticity, including predictive coding frameworks like Bayesian optimal integration [50] and Hebbian synaptic plasticity rules [51], demonstrate how repeated AV training strengthens retinotectal synapses. These models show that spatiotemporally congruent AV inputs induce long-term potentiation (LTP) in SC neurons, consistent with empirical rehabilitation outcomes [19].

Additionally, ipRGCs modulate attention and influence sensory prioritization, acting as key conduits for light’s direct cognitive effects. By engaging neural circuits (e.g., locus coeruleus-mediated alertness pathways), ipRGCs may enhance responsiveness to multisensory stimuli, facilitating timely behavioral responses during heightened arousal and optimizing performance in complex environments [52]. This ipRGC-SC crossmodal link represents an intricate mechanism through which visual cues enhance the processing of other sensory modalities, contributing to coherent perception.

A critical distinction emerges here: while SC multisensory integration is empirically validated (Section 2.1, Section 2.2, Section 2.3), ipRGCs’ role is primarily modulatory (e.g., light-dependent SC priming). Direct ipRGC involvement in crossmodal summation remains hypothetical and warrants targeted investigation.

### 4.4. Implications for Spatial Neglect and Hemianopia

Patients with hemispatial neglect—a disorder characterized by an inability to attend to contralesional space—often exhibit impaired SC function. Research indicates that light significantly influences biological and cognitive functions through ipRGCs, particularly in response to blue-enriched illumination [41,45,53]. This light therapy may improve symptoms of neglect by improving ipRGC-SC signaling, akin to how monocular interventions like eye patching modulate SC activity and eye movements [54]. Additionally, activating melanopsin through light therapy affects visual processing pathways [35,43,55], suggesting its potential as a therapeutic intervention for hemispatial neglect. Clinical evidence supports the efficacy of light therapy in enhancing recovery from hemispatial neglect [56], providing a solid context for these interventions. However, while animal studies suggest ipRGCs prime SC responses to AV stimuli [43,48,51], human behavioral outcomes (e.g., improved spatial attention post-light therapy) are preliminary and may reflect broader circadian or arousal effects [53,56]. Similarly, in hemianopia patients with V1 damage, residual ipRGC projections to the SC may mediate blindsight—the preserved ability to detect light stimuli without conscious visual perception [18]. In contrast, non-conscious detection of neglect likely involves distinct mechanisms [57]. In pediatric hemianopia, AV training enhances oculomotor strategies, likely via SC-mediated plasticity [58]. Given ipRGCs’ projections to the SC, light therapy could further potentiate this pathway by augmenting SC sensitivity to crossmodal cues. Harnessing this pathway through AV rehabilitation—such as pairing light cues with sounds in the blind field—could further promote recovery [31].

### 4.5. Melanopsin Signaling: A Molecular Lever for Rehabilitation

Melanopsin, the photopigment defining ipRGCs, operates via a Gq-coupled signaling pathway, distinct from the phototransduction mechanisms of rods and cones.

#### 4.5.1. The Melanopsin Phototransduction Cascade

**Figure 2:**
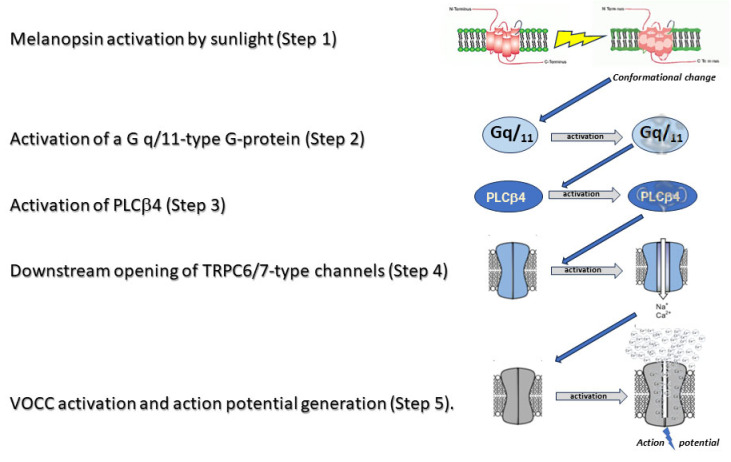
Melanopsin signaling. Melanopsin operates via a Gq-coupled pathway, distinct from rod/cone phototransduction. VOCC: Voltage-operated Calcium Channel [59].

Upon light absorption, melanopsin undergoes a conformational change, activating Gq proteins and triggering intracellular cascades essential for ipRGC photoreception [35,41]. This stimulates phospholipase C beta 4 (PLCβ4), generating inositol trisphosphate (IP3), which mobilizes calcium ions (Ca^2+^) from intracellular stores, altering cellular excitability [39,48]. The resulting Ca^2+^ influx activates TRPC6/7 channels, driving depolarization [44] (Figure 2).

Critically, melanopsin’s Gq-PLCβ4-IP3-TRPC axis sustains ipRGC depolarization—a mechanism divergent from classical photoreceptors and firmly established in animal models [35,44].

#### 4.5.2. Melatonin’s Dual Role: Retinal and Central Modulation

The remarkable ability of ipRGCs to sustain firing rates long after light exposure ends is dynamically regulated by circadian signals, with melatonin emerging as a key modulator. Synthesized rhythmically by the pineal gland, melatonin fine-tunes ipRGC responses primarily through MT1 receptors [60]. This modulation of ipRGC excitability [61,62] reveals melatonin’s dual role in maintaining circadian stability while potentially enhancing visual processing in ways we are only beginning to understand.

During daylight hours, when endogenous melatonin levels are naturally low, experimental administration of melatonin produces interesting effects on M4-type ipRGCs. These specialized cells show broadened depolarizing photoresponses that selectively amplify rod- and cone-driven inputs while leaving intrinsic melanopsin responses unaffected [63]. This selective amplification may represent an elegant mechanism for adapting retinal sensitivity to the subtle light changes characteristic of twilight transitions. As night falls and melatonin levels rise, the system appears to shift priorities—the same hormone that enhanced daytime responses may now dampen ipRGC excitability, favoring robust circadian entrainment over acute visual processing.

This mechanism becomes more complex when we consider melatonin’s actions beyond the retina. In rodents, daytime melatonin suppression of hippocampal clock genes like *Per1* has been shown to enhance long-term potentiation [64], suggesting a possible role in temporally coordinating crossmodal learning. While such mechanisms remain hypothetical in humans, the implications are profound. Melatonin’s local retinal effects, which sharpen photic responses, work in concert with its central actions on synaptic plasticity to create a temporally aligned system where environmental light cues and neural plasticity windows are exquisitely synchronized.

This dual retinal–central modulation positions melatonin as a master regulator of light-dependent adaptation. Through MT1 receptors, it fine-tunes ipRGC excitability [60], while simultaneously influencing hippocampal plasticity [64], effectively bridging the gap between retinal light detection and brain-wide circadian rhythms. The daytime broadening of M4 ipRGC responses to conventional photoreceptor inputs [63] contrasts with the nocturnal suppression of excitability, creating a dynamic system that balances immediate visual needs with long-term circadian requirements. Together, these mechanisms suggest that melatonin’s orchestration of retinal and central processes could optimize rehabilitation approaches by aligning environmental light cues with the brain’s natural plasticity rhythms.

#### 4.5.3. Spatiotemporal Synergy for Rehabilitation

These findings reveal a spatiotemporal partnership where melanopsin encodes environmental light [35,42], while melatonin regulates how and when these signals influence neural plasticity [60,64]—a synergy ripe for rehabilitation. Pairing melanopsin-targeted light therapy with circadian-timed melatonin could thus optimize protocols by aligning photic inputs with endogenous rhythms [56,63].

Though ipRGCs are primarily light-sensitive, their projections to multisensory hubs (e.g., SC) [43,48] and modulation by melatonin [61,62] create conditions conducive to crossmodal integration in downstream nuclei. The sustained depolarization of ipRGCs, enabled by melanopsin’s unique Gq-coupled signaling [35,44], enhances tonic arousal levels [41,52]. Given their role in non-image-forming functions (e.g., circadian entrainment, alertness) [32,37], this response pattern suggests strategic utility in rehabilitation.

Therefore, coordinating melanopsin-driven light detection with melatonin’s circadian modulation may optimize timing-dependent neuroplasticity, particularly in crossmodal rehabilitation paradigms [19,31].

#### 4.5.4. Crossmodal Integration via ipRGC-SC Pathways

By harnessing the sustained firing of ipRGCs following light exposure, rehabilitation programs could potentially enhance attentional focus and cognitive arousal in patients with neurological conditions, thereby improving their overall functional recovery. Such applications highlight the therapeutic potential of targeting melanopsin and ipRGCs through specific light therapy interventions, emphasizing their role in promoting adaptive outcomes in clinical practice [35,41,65]. Indeed, the role of melanopsin in enhancing ipRGC signaling underlines its potential in neurorehabilitation techniques, particularly through its integration with auditory input to facilitate crossmodal recovery, echoing the observed synergy between light and sound stimuli in improving outcomes for patients with visual impairments [18]. Neurocomputational models predict that AV training strengthens retinotectal pathways, even in V1-lesioned patients, by enhancing multisensory integration in the SC [19,31,51]. Given ipRGCs’ ability to prime SC excitability (particularly in low-light conditions: [43,48]) these cells may critically facilitate crossmodal rehabilitation. Therefore, ipRGCs’ tonic arousal effects [52] and SC projections create a bridge between light therapy and multisensory rehabilitation, though human evidence remains limited.

#### 4.5.5. Future Directions and Translational Challenges

The implications of utilizing ipRGC responses in rehabilitation present a promising area for future studies aimed at enhancing recovery in patients with deficits in arousal and attentional capacities. Subtype-specific ipRGC targeting and personalized dosing are critical next steps to translate melanopsin biology into clinical practice.

Therefore, the SC’s layered architecture and multisensory plasticity make it a pivotal target for rehabilitation, bridging early sensory processing and adaptive behavior.

## 5. Therapeutic Potential

Therapeutic interventions targeting melanopsin pathways offer multifaceted approaches for neurorehabilitation, spanning circadian entrainment, multisensory integration, and pharmacological modulation. This section evaluates these strategies—from non-invasive light and AV therapies to emerging melanopsin-targeted drugs—while addressing their mechanisms, clinical potential, and outstanding challenges.

### 5.1. Circadian Interventions: Light and Melatonin Synergy

The therapeutic potential of melanopsin-targeted interventions in neurological rehabilitation presents exciting opportunities for enhancing recovery and cognitive function in various patient populations. One significant avenue of exploration involves circadian interventions that utilize light to stabilize sleep patterns in patients who have experienced strokes. Recent studies have demonstrated that light targeting melanopsin can effectively regulate circadian rhythms, which, in turn, contributes to improved sleep quality [66].

Critically, melatonin may play a dual role in this context. Animal studies suggest that melatonin stabilizes circadian rhythms via the suprachiasmatic nucleus (SCN) while directly priming ipRGCs and enhancing hippocampal plasticity [64]. This has led to the hypothesis of a two-pronged therapeutic strategy: (1) nighttime supplementation to consolidate sleep and (2) targeted daytime administration to enhance learning-dependent rehabilitation (e.g., pairing melatonin with prism adaptation therapy in neglect patients). Preliminary evidence from animal models indicates that melatonin amplifies rod/cone-driven ipRGC responses during daylight [63], which could theoretically boost the efficacy of crossmodal cues in low-light conditions. However, this interaction remains speculative, particularly in humans, and the underlying mechanisms—whether mediated by changes in ipRGC membrane excitability, synaptic input strength, or downstream signal relay—are not yet fully elucidated [41]. Further research is needed to validate these hypotheses and clarify the precise pathways involved.

By optimizing sleep through light exposure, these interventions not only address a fundamental aspect of patient care but also create an indirect pathway for enhancing attentional capacities and overall cognitive performance.

As summarized in Table 3, the interplay between bottom-up (ipRGC-driven) and top-down (cortical) pathways might offer distinct yet complementary therapeutic levers for visual rehabilitation.

### 5.2. Crossmodal Priming for Spatial Neglect

Beyond circadian interventions, crossmodal priming shows promise for rehabilitating hemispatial neglect. Federici et al. [67] recently demonstrated the brain’s capacity for rapid crossmodal plasticity following brief monocular deprivation, where reduced visual input enhanced auditory cortical responses. This compensatory plasticity underscores the potential of multisensory approaches in rehabilitation.

Emerging evidence suggests that blue light (460–490 nm) could augment therapies like prism adaptation by activating ipRGCs and their SC projections [31]. Prism adaptation recalibrates spatial awareness in neglect patients through visuomotor cues [31], and blue light may amplify these effects by priming ipRGC-SC pathways to enhance multisensory integration and contralesional attention. Clinically, controlled blue light exposure (30–100 lux, 30–120 min/day) improves circadian rhythms [53,66] and cognition in stroke patients [68], while AV therapies independently engage SC plasticity [69]. Although exposures under 300 lux are well tolerated [62], optimizing parameters (e.g., intensity, timing) and verifying SC engagement via neuroimaging [70] remain critical.

### 5.3. Optimizing Multisensory Rehabilitation

Stable post-training EEG changes [71] and fMRI evidence of thalamocortical reorganization after AV training [68] suggest that ipRGC-SC pathways may sustain long-term plasticity. Specifically, Alwashmi et al. [68] observed SC and thalamic Blood Oxygen Level-dependent (BOLD) signal modulation during virtual reality-based AV rehabilitation, aligning with computational predictions of retinotectal plasticity [51] and the role of ipRGCs in priming SC excitability [27]. Therefore, combining melanopsin-targeted light with AV cues is a speculative strategy that could optimize rehabilitation timing, pending validation in clinical trials.

Notably, direct evidence for melatonin’s impact on human crossmodal rehabilitation remains limited. While rodent studies demonstrate hippocampal LTP enhancement [64], translational studies must confirm whether daytime melatonin improves multisensory learning in patients. Similarly, the role of ipRGCs in auditory integration, although plausible via SC projections, lacks electrophysiological confirmation.

### 5.4. Pharmacological Modulation: Opportunities and Challenges

Finally, there is emerging interest in the role of pharmacological strategies that leverage the properties of melanopsin. Melanopsin plays a vital role in regulating circadian rhythms across vertebrates, with conserved and species-specific contributions to non-visual phototransduction [72]. Pharmacological modulation of melanopsin signaling, such as through synthetic antagonists like opsinamides, represents a promising frontier for targeted interventions in circadian disorders, photophobia, and other light-dependent behaviors [73]. These compounds can directly inhibit ipRGC activity, reducing melanopsin-mediated light signaling. However, this approach requires careful consideration of potential off-target effects, particularly given melanopsin’s broad role in non-image-forming functions. For instance, systemic suppression of ipRGCs could disrupt circadian entrainment, pupillary reflexes, or mood regulation, underscoring the need for tissue-specific delivery or temporally constrained dosing. Additionally, compensatory plasticity in downstream pathways (e.g., increased reliance on rod/cone inputs) may alter therapeutic efficacy over time. Future development should prioritize subtype-selective melanopsin modulators and rigorous preclinical evaluation of long-term safety, ensuring that interventions preserve essential photic regulation while mitigating pathological light sensitivity. Given their capacity to modulate circadian rhythms and light-dependent behaviors, a compelling question arises: could melanopsin antagonists like opsinamides indirectly influence multisensory plasticity? The implications are significant. If melanopsin suppression alters crossmodal neural processing, this could open new avenues for studying—and potentially rehabilitating—sensory and cognitive deficits. Conversely, melanopsin agonists (if and once developed) could enhance ipRGC activity, thereby strengthening light-driven circadian entrainment and multisensory integration—a strategy with distinct potential for neurorehabilitation.

Table 4 summarizes potential therapeutic approaches leveraging multisensory integration mechanisms.

Together, these findings position melanopsin and melatonin as complementary regulators of rehabilitation-relevant plasticity. Melanopsin provides the ‘where’ and ‘what’ of environmental light, while melatonin determines the ‘when’ of neural adaptation. However, the following critical questions remain: (1) Can timed melatonin synergize with AV training in human patients as it does in rodents? (2) Do ipRGCs directly participate in multisensory convergence or solely enable it via downstream nuclei? Addressing these gaps will refine targeted interventions for visual disorders.

### 5.5. Quantifying Light Stimuli for Rehabilitation

The efficacy of light therapy hinges on precise optical parameters, yet current protocols often lack standardization. To bridge this gap, the following could be recommended.

- Melanopic Equivalent Daylight Illuminance (EDI): Quantify melanopsin activation using standardized metrics (e.g., CIE S 026:2018 [74]) to ensure consistent ipRGC stimulation across studies [53,66].
- Spectral Composition: Report spectral power distributions (380–780 nm) of light stimuli, as melanopsin’s peak sensitivity (~480 nm) can be obscured by broader-spectrum sources [44,53].
- Directional Luminance: Characterize spatial properties (e.g., Ganzfeld vs. directional cues) to differentiate local (e.g., SC-targeted) from global (circadian) effects [55].

These measures would enable cross-study comparisons and clarify dose–response relationships in rehabilitation.

## 6. Final Remarks

**Figure 3:**
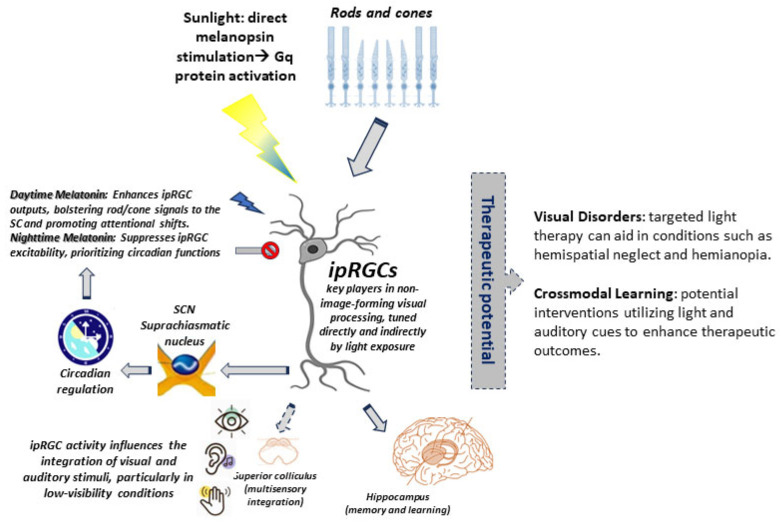
Overview of ipRGC functions and rehabilitation implications. Dotted arrows: proposed. The involvement of ipRGC in multisensory integration and therapeutic potential remains speculative (AV rehab without ipRGC isolation).

In closing, this review presents a transformative perspective on the role of ipRGCs. Traditionally viewed merely as conduits for non-image-forming light perception, we propose that these cells serve as critical regulators of multisensory integration and crossmodal rehabilitation. Unlike conventional models that emphasize cortical and subcortical hubs like the superior colliculus (SC) and parietal cortex in multisensory processing, we argue that ipRGCs, through their unique melanopsin-driven signaling mechanisms and expansive neural projections, may create conditions conducive to adaptive multisensory plasticity, though direct evidence in humans is limited (Figure 3).

Three vital insights emerge from this exploration:

*Multisensory Gatekeepers*: While ipRGCs may not directly integrate non-photic stimuli such as sound or touch, their anatomical projections to multisensory nuclei, particularly the SC, are well documented, although their functional role in crossmodal modulation is inferred from indirect evidence. By influencing SC excitability in response to light cues (as shown by animal studies), ipRGCs might enhance the salience of spatially aligned AV stimuli, although human evidence is still lacking. This positions them as contextual filters that allow environmental light dynamics to shape how downstream circuits process and weight multisensory inputs, thereby optimizing orienting behaviors [27,43].

*Melatonin as a Temporal Lever*: The interplay between the melanopsin and melatonin pathways forms a critical spatiotemporal partnership for rehabilitation outcomes. Daytime secretion of melatonin appears to amplify outputs from rod and cone photoreceptors via ipRGCs [63], while its suppression at night prioritizes circadian rhythm entrainment. This bidirectional regulation suggests a dual therapeutic strategy: employing light therapy to prime the ipRGC-SC pathways, alongside circadian-timed melatonin administration to align reinforcement and neuroplasticity windows with rehabilitation exercises.

*Translational Potential*: The significance of the ipRGC-SC pathway extends into clinical applications, particularly in the treatment of visual disorders. For example, in conditions like hemispatial neglect, targeted blue light that stimulates melanopsin could enhance prism adaptation therapy, sharpening attentional shifts mediated by the SC. Furthermore, in hemianopia patients with V1 damage, residual ipRGC-SC projections may provide a neural substrate for blindsight, potentially enabling rehabilitation through paired AV cues in the blind field. Thus, utilizing ipRGC-driven light therapy, alongside multimodal inputs as evidenced by Purpura et al. [17] and Leo et al. [18], opens new avenues for enhancing recovery in sensory processing disorders, particularly hemianopia and hemispatial neglect. Stable post-training improvements in hemianopia, including P3 amplitude shifts in EEG [71], suggest that ipRGC-SC pathways may sustain long-term plasticity. Combining light therapy with AV cues could exploit this mechanism. Finally, innovative approaches, including melanopsin antagonists [73] and closed-loop light-melatonin protocols, hold promise for personalizing rehabilitation interventions.

### 6.1. Future Directions and Research Gaps

Looking ahead, some future directions warrant consideration.

#### 6.1.1. Mechanistic Clarity

It is crucial to acquire electrophysiological evidence to elucidate whether ipRGCs directly modulate multisensory neurons in the SC or operate via intermediary circuits. Such clarity will enhance our understanding of the functional roles of ipRGCs within the larger multilevel sensory processing system. While AV training’s efficacy is established [69], the role of melanopsin-driven ipRGC inputs to the SC remains unexplored. Targeted light stimulation could optimize the timing and salience of AV cues. The seminal work by Mazziotta et al. [70] demonstrated that AV stimuli induce metabolic changes in primary and associative cortices, with hemispheric specialization for auditory processing. Their findings underscore the importance of multisensory-driven neuroplasticity—a framework that could inform future studies on ipRGC-SC interactions. For instance, combining melanopsin-specific light stimulation with Positron Emission Tomography (PET) in primate models, or employing ipRGC-targeted paradigms in human psychophysical tasks, could help determine whether ipRGCs amplify crossmodal metabolic responses in the SC or higher-order cortices.

#### 6.1.2. Clinical Validation

Research involving human trials is imperative to establish whether daytime melatonin can indeed enhance crossmodal learning, particularly in the context of neglect rehabilitation. Additionally, determining if ipRGC-targeted light therapy produces improvements beyond mere circadian stabilization will be vital. Future studies could use melanopsin antagonists (e.g., opsinamides) to probe the necessity of ipRGC-SC signaling in AV training, while agonists—if developed—might enhance its efficacy. The recent fMRI study by Alwashmi et al. [68] provides direct evidence that AV training in VR augments thalamocortical multisensory integration, correlating with behavioral gains. Their paradigm—a VR adaptation of hemianopia scanning training—could be adapted to test ipRGC contributions by incorporating melanopsin-optimized light cues. For example, pairing their AV stimuli with blue vs. red light (to preferentially activate ipRGCs vs. cones) may dissociate melanopsin’s role in crossmodal plasticity. Their thalamic BOLD signal findings are particularly relevant, as the thalamus receives direct ipRGC projections [48], suggesting a testable link between ipRGC-driven thalamic activation and rehabilitation outcomes.

### 6.2. Project Proposal

**Figure 4:**
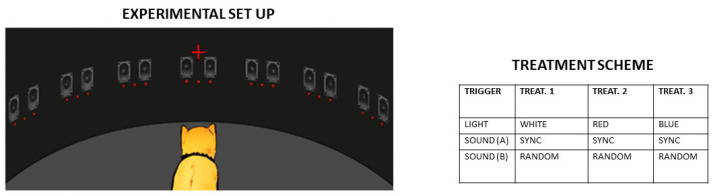
Experimental setup and treatment scheme. Experimental subjects with hemianopia or visual neglect are posed in front of a screen, with the head fixed so that the gaze is directed to the center of the screen. Then, lights of different wavelengths coupled to audible sounds coming from the very same or opposite directions are proposed in a fixed or random sequence. The training (lasting 15 min) will be repeated 2 to 3 times/day for at least one month, after which the perception in the blind area will be measured by electrophysiological means, and by behavioral reactions. Left: experimental setup. Right: treatment groups (each group composed of 3 animals, either wild type or K.O. for melanopsin expression; controls receive no treatment). Once the results of this first part are determined, melatonin effects will be addressed under chosen conditions.

To improve our understanding of the different pathways involved in visual rehabilitation, a project could be proposed, utilizing a validated animal model of monocular visual deprivation. This experiment will test whether ipRGCs mediate AV integration in a controlled model, providing foundational data for future human trials. Specifically, the aim is to address the role of multisensory ganglion cells responsive to both sound and light. This study will investigate how the combination of directional light and sound stimuli impacts the visual response of the injured animal, using a screen equipped with both light and sound signals (Figure 4).

This setup will allow us to explore the efficacy of different wavelengths (e.g., blue vs. red light) in eliciting a visual response from the subjects. The role of ipRGCs in this process will be investigated using animals in which the genes for melanopsins have been knocked out. Furthermore, this study will analyze the effects of melatonin given as an oral supplement at different times of the day (morning vs. evening) to determine whether such treatment influences the rehabilitation response of the animal.

Mazziotta’s metabolic maps [70] could guide target region analysis (e.g., SC, thalamus) in animal models, while Alwashmi’s [68] VR-based AV training protocol offers a translational bridge to human trials—should preliminary results prove promising. Encouraging findings would then justify clinical studies in humans with amblyopia or hemianopia, verifying whether similar ipRGC-mediated mechanisms enhance rehabilitation. This cross-species validation would refine patient-specific strategies, leveraging animal-derived insights to optimize multisensory rehabilitation protocols.

### 6.3. Limitations and Unanswered Questions

The promising role of ipRGCs in multisensory rehabilitation outlined in this review must be tempered by several critical limitations that highlight the gap between animal research and human clinical applications. At present, our mechanistic understanding relies overwhelmingly on rodent studies—from the foundational work mapping ipRGC projections to the superior colliculus [48] to detailed analyses of melanopsin phototransduction [35]. While these animal models have been indispensable, the leap to human physiology remains precarious. Our current human evidence is frustratingly indirect, limited to fMRI correlations [68] and behavioral outcomes [56] that cannot disentangle ipRGC-specific effects from broader circadian or arousal influences. This glaring absence of direct electrophysiological evidence in humans, particularly recordings demonstrating ipRGC modulation of SC multisensory neurons, represents perhaps the most pressing void in the field.

The contrast between established mechanisms and unproven therapies creates a translational chasm. On one hand, rodent studies have convincingly demonstrated the anatomical reality of ipRGC-SC connections [27,48] and clarified melanopsin’s role in non-image-forming vision [42]. Yet proposed clinical interventions—like combining blue light therapy with AV training [31]—remain frustratingly speculative. The field lacks crucial evidence that ipRGC activation can selectively enhance crossmodal plasticity in patients, rather than simply producing generic alertness effects that might be achieved through any stimulating intervention.

Several fundamental questions must guide future research to bridge this divide. First and foremost is the need to clarify whether ipRGCs directly modulate SC multisensory neurons or exert their influence indirectly through thalamocortical relays [27]. The timing of interventions presents another crucial unknown—can melanopsin-targeted approaches synergize with AV training in a circadian-dependent manner, as suggested by melatonin’s biphasic effects [63]? Species differences complicate the picture further, as we still do not know whether human ipRGC subtypes mirror their rodent counterparts in their projection patterns and functional roles [33]. Even the basic parameters of light therapy—melanopic EDI, chromatic contrast, and temporal dynamics—require systematic investigation to determine their impact on rehabilitation efficacy.

Addressing these questions will demand innovative approaches across multiple research fronts. Primate studies combining melanopsin-specific light stimulation with direct SC recordings could help bridge the animal–human divide. Human psychophysical paradigms must be developed to tease apart ipRGC contributions from classical photoreceptor inputs. Most critically, properly controlled clinical trials are needed to evaluate whether ipRGC-targeted interventions offer benefits beyond circadian regulation and nonspecific arousal effects. Until these studies are conducted, the therapeutic potential of ipRGC modulation will remain more promise than reality.

### 6.4. Conclusions

This synthesis of retinal biology and multisensory neuroscience reveals a paradigm shift in understanding visual processing. The retina, far from being a mere passive sensor, emerges—through ipRGCs—as an active orchestrator of crossmodal plasticity. Melanopsin-expressing ganglion cells form a biological bridge between environmental light and brain-wide sensory integration, dynamically modulating how we perceive and interact with multisensory stimuli.

The therapeutic implications are profound. By strategically leveraging ipRGCs’ unique capacity to combine photic input with circadian timing, we may pioneer novel rehabilitation approaches that synergize light, temporal biology, and multisensory experience. Such strategies could transform outcomes for patients with sensory processing deficits, from hemispatial neglect to post-stroke visual impairments.

Yet, this promise remains tempered by critical unknowns. While animal studies illuminate ipRGCs’ anatomical and molecular foundations [35 Berson, 48 Hattar], human applications require rigorous validation. Key questions persist: Can we selectively engage ipRGC pathways to enhance crossmodal plasticity without disrupting essential non-visual functions? Do melanopsin-targeted interventions offer measurable advantages over conventional therapies?

The path forward demands interdisciplinary collaboration—bridging retinal electrophysiology, systems neuroscience, and clinical rehabilitation—to translate these insights into evidence-based practice. Only through such integration will we determine whether ipRGC-mediated approaches represent a true therapeutic breakthrough or an intriguing but limited adjunct to existing modalities.

## Figures and Tables

**Table 1:** Primary divisions of the optic pathway. Optic nerve fibers diverge into distinct pathways, targeting different brain structures based on their functional roles.

**Optic Nerve**	**Pathways**	**Targets**	**Fibers**	**Function**
	Image Forming (conscious vision)	Lateral Geniculate Nucleus (LGN) → Primary Visual Cortex (V1)	~90% of RGC axons (mostly from rods/cones).	High-acuity, conscious visual perception (shape, color, motion).
	Non-Image Forming (Reflexive/Circadian)	Superior Colliculus (SC)	~10% of RGCs (including ipRGCs and motion-sensitive RGCs)	Orienting reflexes, multisensory integration, and spatial attention
		Pretectal Nuclei	Subset of RGCs (mainly ipRGCs and ON cells)	Pupillary light reflex and accommodation
		Suprachiasmatic Nucleus (SCN)	Dedicated ipRGCs (melanopsin-expressing)	Circadian rhythm entrainment
		Ventral Lateral Geniculate (vLGN) & Intergeniculate Leaflet (IGL)	Non-image-forming RGCs	Modulation of circadian rhythms by non-photic cues (e.g., activity)

**Table 2:** SC neuron classification based on their response.

Type	Description	References
Visual Responsive Neurons	These neurons respond to visual stimuli. The superior colliculus receives direct input from the retina and is involved in processing visual information related to the location and movement of objects in the visual field	[23]
Auditory Responsive Neurons	Neurons in the superior colliculus respond to auditory stimuli. These neurons contribute to the localization of sound sources in the environment.	[24,25]
Somatosensory Responsive Neurons	Some neurons in the superior colliculus are responsive to somatosensory stimuli, such as touch or proprioception. This input helps in integrating tactile and proprioceptive information with visual and auditory signals.	[22,26]
Multisensory Neurons	Multisensory neurons are those that integrate information from more than one sensory modality. These neurons play a critical role in combining signals from different senses to create a more accurate and robust representation of the external world.	[2,10,22]

**Table 3:** Bottom-up (ipRGC-driven) vs. top-down (cortical) pathways in SC plasticity: mechanisms and therapeutic implications. Bottom-up pathways prime SC excitability, while top-down pathways refine spatial maps. Combined protocols (e.g., morning light + afternoon prism training) may maximize recovery.

Feature	Bottom-Up (ipRGC-Driven Pathways)	Top-Down (Cortical Feedback Pathways)
Stimulus Origin	Light (480 nm blue light), auditory/somatosensory cues	Cognitive demands (attention, predictions), learned spatial statistics
Key Structures	- Retina (ipRGCs) - Superior colliculus (SC) - Inferior colliculus (IC)/brainstem	- Frontal eye fields (FEF) - Parietal cortex - Thalamus
Neural Pathways	ipRGCs → SC (direct melanopsin projections) + IC/somatosensory → SC convergence	FEF/parietal cortex → thalamus → SC (corticotectal feedback)
Mechanisms	- Melanopsin/TRPC depolarization - Sustained firing - Inverse effectiveness (multisensory)	- Alpha (8–12 Hz) oscillations (attention) - Theta (3–7 Hz) oscillations (conflict resolution)
Temporal Dynamics	Tonic (slow, sustained)	Phasic (fast, task-dependent)
Functional Outcomes	- Reflexive orienting (e.g., gaze shifts) - Blindsight recovery	- Voluntary visual search - Prism adaptation (spatial recalibration)
Clinical Targets	Hemianopia, low-light rehabilitation	Spatial neglect, attentional deficits
Therapeutic Levers	- Blue light therapy - Melanopsin agonists - AV cue timing	- Prism adaptation - Cognitive training - Transcranial stimulation

**Table 4:** Therapeutic potential.

Strategy	Actuation	References
Circadian Optimization	Light therapy targeting melanopsin stabilizes sleep patterns in stroke patients, improving cognitive recovery	[66]
	Melatonin supplementation complements the above: nighttime doses consolidate sleep, while daytime administration enhances learning-dependent plasticity via MT1 receptors in ipRGCs and hippocampus	[63,64]
Crossmodal Priming	In hemispatial neglect, pairing blue light (activating ipRGC-SC pathways) with prism adaptation therapy may amplify attentional shifts to the neglected field.	[31]
	Federici and colleagues demonstrate that auditory crossmodal plasticity can compensate for visual deprivation, suggesting synergistic potential with light/melatonin timing.	[67]
Pharmacological Modulation	Melanopsin antagonists (e.g., opsinamides) which selectively inhibit melanopsin-mediated light responses highlights the potential for targeted interventions in circadian disorders, photophobia, and other light-dependent behaviors.	[73]

## Abbreviations

The following abbreviations are used in this manuscript:

**Abbreviation**	**Full Form**
AV	Audiovisual
BOLD	Blood Oxygen Level Dependent
Ca^2+^	Calcium Ions
CC	Creative Commons (License)
CIE	International Commission on Illumination (French: Commission Internationale de l’Éclairage)
EDI	Equivalent Daylight Illuminance
EEG	Electroencephalogram
FEF	Frontal Eye Field
fMRI	Functional Magnetic Resonance Imaging
Gq	Gq Protein (a type of G-protein)
IC	Inferior Colliculus
IGL	Intergeniculate Leaflet
IP3	Inositol Trisphosphate
ipRGCs	Intrinsically Photosensitive Retinal Ganglion Cells
LGN	Lateral Geniculate Nucleus
LTP	Long-term Potentiation
MT1/MT2	Melatonin Receptor Type 1/Type 2
NB	Nota Bene (“Note Well”)
PET	Positron Emission Tomography
PLCβ4	Phospholipase C beta 4
RGCs	Retinal Ganglion Cells
SC	Superior Colliculus
SCN	Suprachiasmatic Nucleus
SOC	Superior Olivary Complex
TRPC6/7	Transient Receptor Potential Canonical 6/7 (channels)
VOCC	Voltage-operated Calcium Channels
VR	Virtual Reality
